# Investigating citrullinated proteins in tumour cell lines

**DOI:** 10.1186/1477-7819-11-260

**Published:** 2013-10-07

**Authors:** Zhongmin Jiang, Yazhou Cui, Lin Wang, Yan Zhao, Suhua Yan, Xiaotian Chang

**Affiliations:** 1 Medical Research Center of Qianfoshan Hospital, Shandong University, Jingshi Road 16766, Jinan, Shandong 250014, P.R. China; 2 Biomedical Research Center, Shandong Academy of Medical Sciences, Jingshi Road 18877, Jinan, Shandong 250062, P.R. China

**Keywords:** Citrullination, Peptidylarginine deiminases, Tumour

## Abstract

**Background:**

The conversion of arginine into citrulline, termed citrullination, has important consequences for the structure and function of proteins. Studies have found PADI4, an enzyme performing citrullination, to be highly expressed in a variety of malignant tumours and have shown that PADI4 participates in the process of tumorigenesis. However, as citrullinated proteins have not been systematically investigated in tumours, the present study aimed to identify novel citrullinated proteins in tumours by 2-D western blotting (2-D WB).

**Methods:**

Two identical two-dimensional electrophoresis (2-DE) gels were prepared using extracts from ECA, H292, HeLa, HEPG2, Lovo, MCF-7, PANC-1, SGC, and SKOV3 tumour cell lines. The expression profiles on a 2-DE gel were trans-blotted to PVDF membranes, and the blots were then probed with an anti-citrulline antibody. By comparing the 2-DE profile with the parallel 2-D WB profile at a global level, protein spots with immuno-signals were collected from the second 2-DE gel and identified using mass spectrometry. Immunoprecipitation was used to verify the expression and citrullination of the targeted proteins in tumour cell lines.

**Results:**

2-D WB and mass spectrometry identified citrullinated α-enolase (ENO1), heat shock protein 60 (HSP60), keratin 8 (KRT8), tubulin beta (TUBB), T cell receptor chain and vimentin in these cell lines. Immunoprecipitation analyses verified the expression and citrullination of ENO1, HSP60, KRT8, and TUBB in the total protein lysates of the tumour cell lines.

**Conclusions:**

The citrullination of these proteins suggests a new mechanism in the tumorigenic process.

## Background

Citrullination, or deimination, is the post-translational modification of arginine into citrulline. This conversion has important consequences for the structure and function of proteins because arginine is positively charged at a neutral pH and citrulline is uncharged. Accordingly, citrullination increases the hydrophobicity of a protein, leading to alterations in protein folding [1]. Arginine residues frequently function as ligand recognition sites in proteins, and some enzymes that interact with negatively charged substrates or cofactors have an arginine residue as an anion recognition site [2]. Therefore, it is highly possible that citrullination participates in regulating the activities of these enzymes. In addition, it has been suggested that citrullination modifies the action of trypsin-like enzymes and trypsin inhibitors, interferes with intermediate filament assembly, and plays a role in rapid cellular turnover in tissues with secretory activity [3-5].

Citrullination is performed by enzymes called peptidylarginine deiminases (PADs). Genes encoding PAD family members, including PADI1, PADI2, PADI3, PADI4, and PADI6, cluster at human chromosome position 1p36.13, and the expression of each of these isoforms has been detected in numerous tissues [6]. Studies have reported that PADs are involved in cell differentiation, apoptosis, nerve growth, embryonic development, and gene regulation [7,8].

Cancer is one of the leading causes of death in humans. In recent years, studies have found that PADI4 is markedly over-expressed in a majority of human cancers, suggesting that PADI4 is a putative target for cancer treatment [9-11]. We recently also reported that the PADI4 gene may be a valid susceptibility gene for oesophageal carcinoma [12]. Cherrington et al. recently detected the unique expression of PADI2 in human mammary gland epithelial cells and MCF-7 breast cancer cells [13]. Therefore, the identification of citrullination substrates is helpful for understanding the pathogenic mechanisms of PADs during tumorigenesis. Thus far, transcription factors, coregulators, and histones have been found to be functional targets for citrullination by PADs, with the citrullination of these targets affecting gene expression in multiple tumour cell lines [14]. Yao et al. and Li et al. found that citrullination and methylation modifications are antagonistic to each other, indicating a conserved posttranslational regulatory strategy [15,16]. Li et al. also demonstrated an important crosstalk between histone deacetylation and citrullination, suggesting a combination of PADI4 and histone deacetylase 2 inhibitors as a potential strategy for cancer treatment [17]. Tanikawa et al. detected the *in vivo* and *in vitro* citrullination of the arginine 3 residue of histone H4 in response to DNA damage through the p53-PADI4 pathway. These authors also showed that the ectopic expression of PADI4 leads to chromatin decondensation and promotes DNA cleavage [18]. Guo et al. used a high-density protein array as a primary screen to identify 40 previously unreported PADI4 substrates, reporting that PADI4 citrullinates the Arg-Gly repeat region of 40S ribosomal protein S2, which is also an established site for Arg methylation by protein arginine methyltransferase 3 [19]. Post-translational modifications of the extracellular matrix are also key events in cancer progression: it has been confirmed that the citrullination of cytokeratin, antithrombin, fibronectin, and ADAMTS4 (a disintegrin and metalloproteinase with thrombospondin motifs-4) is involved in abnormal apoptosis, high coagulation properties, and disordered cell proliferation and differentiation, all of which are main features of malignant tumours [20-25]. Therefore, the precise knowledge of citrullination modifications of a protein can provide invaluable information regarding the aetiological importance of these citrullinated proteins.

Although several proteins, such as filaggrin, fibrin, and vimentin are confirmed substrates of PADs, citrullinated proteins in tumours have not been systematically investigated to date. In the present study, we aimed to identify novel citrullinated proteins in tumours using a proteomic method and 2-D western blotting (2-D WB). Because tumour tissues contain both tumour cells and normal cells, and because tissue structures develop with the progress of pathophysiological conditions, we screened the citrullinated proteins in various cultured tumour cell lines. This method is regarded by many as a sound approach to effectively identify common features of tumour tissues under variable conditions.

We employed two identical, two-dimensional electrophoresis (2-DE) gels per experiment to analyse several tumour cell lines and then trans-blotted the expression profiles of one of the 2-DE gels to a polyvinylidene fluoride (PVDF) membrane to perform western blotting (WB) by probing with an anti-citrulline antibody. By comparing the expression profiles of the 2-DE gel to the results of the parallel WB, the protein spots recognised by an immuno-signal were collected from the 2-DE gel and identified using mass spectrometry. The proteins that were recognised by the anti-citrulline antibody were regarded as citrullinated proteins in the tumour cell lines. Immunoprecipitation was performed to verify the citrullination of the targeted proteins in the tumour cell lines.

## Methods

### Cell cultures

ECA (originating from oesophageal carcinoma), HEPG2 (originating from liver cancer), SKOV3 (originating from ovarian cancer), MCF-7 (originating from breast cancer), H292 (originating from lung cancer), HeLa (originating from cervical cancer), Lovo (originating from colon cancer), OS-RC (originating from kidney cancer), PANC-1 (originating from pancreatic cancer), and SGC (originating from gastric cancer) were cultured in Dulbecco’s modified Eagle’s medium supplemented with 10% foetal calf serum, 50 U/mL penicillin, and 50 μg/mL streptomycin in an atmosphere of 5% CO_2_ at 37°C.

### Two-dimensional electrophoresis and two-dimensional western blot analysis

The samples of the cultured cells were collected and homogenised in lysis buffer (7 M urea, 2 M thiourea, 4% CHAPS, 2% IPG buffer, 65 mM DTT, and 1 mM PMSF) with Protease Inhibitor Cocktail (Sigma, USA) on ice and then centrifuged at 14,000 × *g* for 30 min.

Isoelectric focusing (IEF) was performed identically twice using an Ettan IPGphor II IEF system (GE Healthcare, USA) with Immobiline Drystrips (7 cm, NL, pH 3–10) (GE Healthcare). After rehydration, 100 μg of protein was rehydrated with an appropriate quantity of rehydration buffer [7 M urea, 2 M thiourea, 4% CHAPS, and 0.5% IPG buffer, pH 3–10 (GE Healthcare), with 0.001% bromophenol blue]. The IEF voltage was applied according to the following protocol: 30 V for 12 h, 150 V for 2 h, 3,000 V for 30 min, 5,000 V for 30 min, linear ramping to 8,000 V over 1 h, and then 8,000 V until 10,000 V-h was reached. Prior to the second dimension separation, the strips were equilibrated in two successive buffers (6 M urea, 2% SDS, 50 mM Tris–HCl, pH 8.8, 30% glycerol, and a trace of bromophenol blue). The first buffer contained 1% w/v DL-dithiothreitol, and the second contained 2.5% w/v iodoacetamide. Each equilibration was conducted for 15 min with continuous agitation. The strips were then rinsed in electrophoresis buffer (25 mM Tris base, 192 mM glycine, and 0.1% w/v SDS), applied to 12.5% acrylamide gels (80 mm × 70 mm), and sealed with melted agarose (0.5% w/v agarose in electrophoresis buffer containing a trace of bromophenol blue). Electrophoresis was conducted using the Mini PROTEAN Tetra Cell (Biorad, USA) with an initial separation at 80 V for 10 min, followed by 200 V at 25°C until the dye front had migrated to the bottom.

After electrophoresis, one gel was visualised by staining with Coomassie brilliant blue R350 staining solution overnight. The gel was scanned with a UMAX Powerlook 2100XL (UMAX Technologies, USA), and the digitised images were analysed with ImageMaster 2D Platinum software 5.0 (GE Healthcare).

The proteins on another gel were trans-blotted onto a PVDF membrane using a Mini-PROTEAN® 3 vertical electrophoresis chamber (Biorad) following the manufacturer’s instructions to conduct a 2-D WB analysis. The membrane was probed with an anti-citrulline antibody (Abcam, USA) that was raised with conjugated citrulline glutaraldehyde protein in rabbits. The antibody specificity was verified with an ELISA. This polyclonal antibody recognises only citrulline residues on proteins and not free citrulline. All of the primary and secondary antibodies were diluted in 5% non-fat dry skim milk in TBST (0.02 M Tris base and 0.137 M NaCl in distilled water, pH 7.6, containing 0.1% Tween 20). The immuno-reactive signals were detected with alkaline phosphatase-conjugated secondary antibodies and visualised using Western Blotting Luminol Reagent (Amersham, USA). The western blot images were acquired on a Typhoon Trio (GE Healthcare).

The 2-DE gel stained with Coomassie blue was compared to the corresponding western blot results. The protein spots in the same positions as the immuno-signals were excised manually from the stained gels, washed in MilliQ water, de-stained in 50 mM ammonium bicarbonate/50% acetonitrile (ACN) buffer, dehydrated in 100% ACN for 15 min, and then completely dried by vacuum centrifugation. The dried gel pieces were suspended in trypsinisation buffer (10 ng/μL trypsin and 25 mM NH_4_HCO_3_, pH 8.0) for 30 min at 4°C and incubated overnight in 10 mL 25 mM NH_4_HCO_3_ at 37°C. Lastly, the peptides were extracted with 5% trifluoroacetic acid (TFA)/50% ACN (v/v), and the extract was lyophilised and re-dissolved in 0.1% TFA/30% ACN (v/v).

### Matrix-assisted laser desorption/ionisation time-of-flight/time-of-flight mass spectrometry (MALDI-TOF/TOF MS) analysis and protein identification

A total of 0.5 μL of peptide sample was mixed with an equal volume of 5 mg/mL α-cyano-4-hydroxycinnamic acid matrix (Bruke, Sweden) in 0.1% TFA/50% ACN and spotted onto a standard 192-well plate (ABI). Six external standards (mass standard kit for the 4700 Proteomics Analyzer calibration mixture, ABI) were used to calibrate each spectrum to a mass accuracy within 50 ppm. The mass spectrometry analysis was performed using an ABI 4700 Proteomics Analyzer MALDI-TOF/TOF mass spectrometer (ABI) using a batch-mode acquisition method. The spectral data were analysed with the international protein index human database v3.10 (57,478 sequences and 25,254,519 residues) using GPS explorer TM software version 3.0 and Mascot 2.0 software (Matrix Science, UK). Protein identification was completed using Mascot software, the benchmark for the identification, characterisation, and quantitation of proteins using mass spectrometry data. The Mascot search parameters were as follows: database, Swiss-Prot; fixed modifications, carbamidomethy; peptide tol, 0.15 kDa; up to 1 missed cleavage allowed.

### Immunoprecipitation

The total proteins were purified from the cultured ECA, PANC-1, SKOV3, HeLa, SGC, and HEPG2 cells using a Total Protein Extraction kit (Biochain, USA) in accordance with the manufacturer’s instructions. The α-enolase (ENO1), cytokeratin 8 (KRT8), heat shock protein (HSP60), protein disulphide isomerase (PDI), T cell receptor beta chain (TCR β), tubulin B (TUBB), and vimentin (VIME) proteins in the samples were immunoprecipitated using a Protein G Immunoprecipitation kit (Sigma) in accordance with the manufacturer’s instructions. The lysates were incubated with an anti-HSP60 antibody, anti-PDI antibody, anti-ENO1 antibody, anti-TCR β antibody, anti-VIME antibody, anti-KRT8 antibody, or anti-TUBB antibody overnight at 4°C. All antibodies were commercially produced and obtained from Abcam. Protein G beads provided with the kit were added to the mixtures and incubated for 2 h at 4°C. After a thorough wash, the extracts were eluted with 1X Laemmli sample buffer (Sigma). The concentrations of the immunoprecipitates were determined using the BCA Protein Assay kit (Pierce, USA). A total of 5 μg of protein sample was separated by SDS-PAGE and trans-blotted onto PVDF membranes using a western blotting apparatus. Two membranes were prepared using the same protocol: one of the membranes was probed with antibodies against ENO1, KRT8, HSP60, PDI, TCR β, VIME, and TUBB, and the other was hybridised with the anti-citrulline antibody (Abcam). Immuno-reactive signals were detected with alkaline phosphatase-conjugated secondary antibodies and visualised using Western Blotting Luminol Reagent (Amersham). The images of the western blots were acquired using a Typhoon Trio (GE Healthcare).

The Ethics Committee of Shandong Provincial Qianfoshan Hospital approved this study.

## Results

### Screen of citrullinated proteins in tumour cell lines by 2-D western blotting

**Figure 1:**
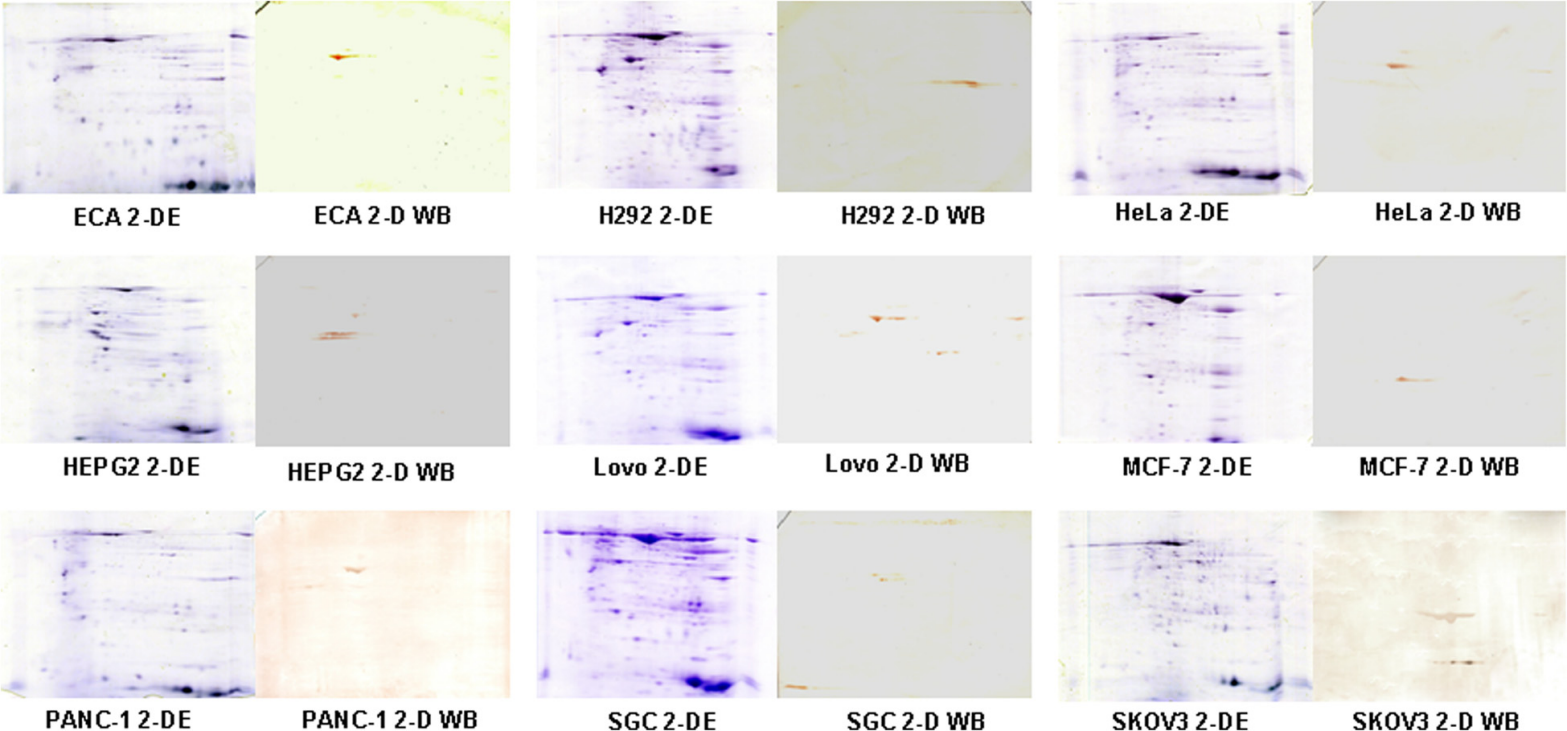
**Two-dimensional polyacrylamide gel electrophoresis and the parallel 2-D western blotting of total proteins from various tumour cell lines.** The total proteins extracted from ECA, H292, HeLa, HEPG2, Lovo, MCF-7, PANC-1, SGC, and SKOV3 cells were separated using two identical 2-DE gels. The tumour cell proteins on one gel were trans-blotted onto a PVDF membrane and probed with an anti-citrulline antibody. The protein spots in the same position as those recognised by the parallel 2-D WB were selected and identified by MALDI-TOF MS.

Following our computational analysis, 200–300 spots were visualised on each 2-DE gel loaded with total proteins from various tumour cell lines. In the present study, 263 spots were detected on the 2-DE gels loaded with proteins from ECA cells, 251 spots from H292 cells, 296 spots from SGC cells, 270 spots from SKOV3 cells, 284 spots from HeLa cells, 255 spots from HEPG2 cells, 235 spots from Lovo cells, 244 spots from MCF-7 cells, and 222 spots from PANC-1 cells. The expression patterns of the 2-DE gels were compared with the expression profiles of the parallel 2-D WBs at a global level. One protein spot recognised by the anti-citrulline antibody was detected in the total protein of the ECA cells in addition to 2 spots from H292 cells, 1 spot from HeLa cells, 3 spots from HEPG2 cells, 1 spot from Lovo cells, 2 spots from MCF-7 cells, 2 spots from PANC-1 cells, 3 spots from SGC cells, and 1 spot from SKOV3 cells. The 2-DE gels and their corresponding western blots are shown in Figure 1. The experiments were repeated three times, and the results of the 2-DE gel and 2-D WB were highly reproducible. The proteins immuno-stained with the anti-citrulline antibody were collected from the gels and analysed by MALDI-TOF/TOF MS. The identified proteins with scores over 70 are shown in Table 1 and were selected for further study. The mass spectrometry analysis yielded the following: ENO1 was detected in the HEPG2, PANC-1, and SGC cells; HSP60 was detected in the ECA, HeLa, and SKOV3 cells; TCR β was detected in the HEPG2, PANC-1, and SGC cells; VIME was detected in the HEPG2 and PANC-1 cells; PDI was detected in the H292 cells; TUBB was detected in the SGC cells; and KRT8 was detected in the MCF-7 cells. No evident immuno-staining was observed on the OR-SC, PC3, or U2-OS cell WBs.

**Table 1:** Identified citrullinated proteins in cultured tumour cell lines using 2-D western blotting

**Spots in cell lines**	**MALDI-TOF/TOF MS analysis**	**Identified proteins**
ECA 3a	Mass: 61187 Score: 133 Expect: 1.2e-08 Matches: 17	60 kDa heat shock protein (HSP60)
H292 6a	Mass: 54454 Score: 104 Expect: 9.2e-06 Matches: 11	Protein disulfide isomerase (PDI)
HeLa 1a	Mass: 60813 Score: 76 Expect: 0.054 Matches: 6	60 kDa heat shock protein (HSP60)
HEPG2 2a	Mass: 50240 Score: 155 Expect: 7.3e-11 Matches: 22	Alpha-enolase (ENO1)
HEPG2 3a	Mass: 36628 Score: 102 Expect: 1.5e-05 Matches: 12	T cell receptor beta chain (TCRb)
HEPG2 6a	Mass: 53738 Score: 149 Expect: 2.9e-10 Matches: 18	Vimentin (VIME)
Lovo 1a	Mass: 48083 Score: 105 Expect: 7.3e-06 Matches: 9	Mitochondrial ATP synthase, H + transporting F1 complex beta subunit
MCF-7 3a	Mass: 53774 Score: 130 Expect: 2.3e-08 Matches: 12	Cytokeratin 8 (KRT8)
PANC-1 1a	Mass: 53738 Score: 140 Expect: 2.3e-09 Matches: 17	Vimentin (VIME)
PANC-1 4a	Mass: 47481 Score: 120 Expect: 2.3e-07 Matches: 13	Alpha-enolase (ENO1)
PANC-1 4a	Mass: 36628 Score: 79 Expect: 0.027 Matches: 9	T cell receptor beta chain (TCRb)
SGC 1a	Mass: 50177 Score: 70 Expect: 0.026 Matches: 10	Tubulin beta (TUBB)
SGC 2a	Mass: 36628 Score: 113 Expect: 1.2e-06 Matches: 11	T cell receptor beta chain (TCRb)
SGC 3a	Mass: 47481 Score: 172 Expect: 1.5e-12 Matches: 15	Alpha-enolase (ENO1)
SKOV 3a	Mass: 61187 Score: 103 Expect: 1.2e-05 Matches: 14	60 kDa heat shock protein (HSP60)

### Verification of citrullination of the candidate proteins in the cell lines by immunoprecipitation

**Figure 2:**
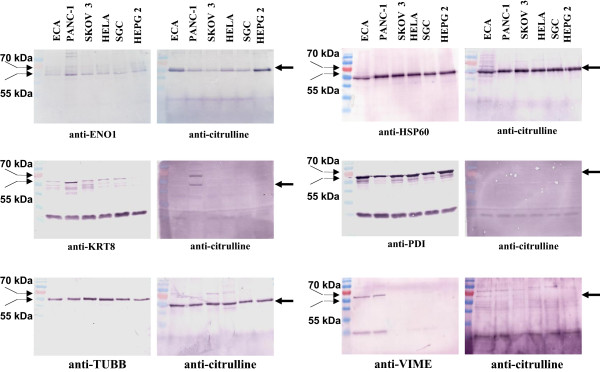
**Immunoprecipitation of ENO1, HSP60, KRT8, PDI, TUBB, and VIME in ECA, HEPG2, SKOV3, HeLa, PANC-1, and SGC cell lines.** The immunoprecipitation was performed with anti-ENO1, -HSP60, -KRT8, -PDI, -TUBB, or -VIME antibodies to extract the corresponding proteins. Western blotting was performed with anti-ENO1, -HSP60, -KRT8, -PDI, -TUBB, -VIME, and anti-citrulline antibodies to determine the citrullination of these candidate proteins. The thick arrow indicates the candidate proteins. The thin arrow indicates the molecular weights of the protein marker.

To verify the above 2-D WB results, immunoprecipitation experiments were performed using antibodies against ENO1, HSP60, KRT8, PDI, TCR β, TUBB, and VIME in the ECA, PANC-1, SKOV3, HeLa, SGC, and HEPG2 cells. ENO1 (53 kDa), HSP60 (60 kDa), and TUBB (55 kDa) were immunoprecipitated from the total protein extracts of ECA, PANC-1, SKOV3, HeLa, SGC, and HEPG2 cells and detected using western blotting with the anti-citrulline antibody. KRT8 (53 kDa) was immunoprecipitated from the total protein extracts of ECA, PANC-1, SKOV3, HeLa, SGC, and HEPG2 cells and was only detected in the PANC-1 cell lysate by western blotting using the anti-citrulline antibody. PDI (58 kDa) was immunoprecipitated from the total protein extracts of ECA, PANC-1, SKOV3, HeLa, SGC, and HEPG2 cells, but the protein was not detected in these tumour cell lines by western blotting using the anti-citrulline antibody. VIME (57 kDa) was immunoprecipitated from the total protein extracts of ECA and PANC-1 cells; although the extracted protein was recognised in these two tumour cell lines, there were multiple immuno-signals using the anti-citrulline antibody. TCR β was not extracted from the total protein extracts of ECA, PANC-1, SKOV3, HeLa, SGC, and HEPG2 cells and was not detected by western blotting using the anti-citrulline antibody. The immunoprecipitation results are shown in Figure 2 and were highly reproducible in three repeat experiments.

## Discussion

In the present study, we conducted a 2-D WB analysis with cultured tumour cell lines. Western blotting was performed with an anti-citrulline antibody, and the protein spots in the same locations as the immuno-signals detected by WB were identified using mass spectrometry. ENO1, HSP60, KRT8, PDI, TCRβ, TUΒΒ, and VIME were identified in the tumour cell lines using 2-D WB, and immunoprecipitation verified that ENO1, HSP60, KRT8, and TUBB were expressed and citrullinated in these cell lines, as described above. These results indicate that ENO1, HSP60, KRT8, and TUBB are novel proteins with citrullination in tumour tissues.

From the results, it can be observed that the amount of citrullinated proteins identified by 2-D WB was much reduced in comparison to those that were immunoprecipitated. Immunoprecipitation is a technique for pulling a protein antigen out of solution using an antibody that specifically binds to that particular protein. Therefore, citrullinated proteins are accumulated by immunoprecipitation, and the quantity of citrullinated protein in the experiment is much more than that observed by 2-D WB. The citrullinated proteins identified by 2-D WB differed greatly in the different cell lines used in this study, as these lines originate from different tissues. Although citrullination is common in tissues, the physiological role and pathogenic mechanism of the citrullinated protein may be different in different tissues or at different pathological stages. In addition, this study used Immobiline Drystrips 7 cm in length, and a 100-μg protein sample was loaded onto the gel; thus, it is reasonable to obtain 200–300 spots in a 7-cm gel length.

Functional analyses have shown that ENO1 is an enzyme, HSP60 is involved in cell signalling pathways, and KRT8 and TUBB are important components of the cytoskeleton. These citrullinated proteins could play similar roles in tumorigenic pathways in different cell lines, though the detailed mechanism needs further investigation. ENO1, a homodimeric, soluble protein, functions in anaerobic metabolism under hypoxic conditions and acts as a cell surface plasminogen receptor during tissue invasion. The irregular expression of ENO1 has been linked to tumour progression in several cases of breast and lung cancer, and the ENO1 protein level in the plasma of non-small cell lung cancer patients was significantly higher than that in the plasma of healthy individuals [26]. Furthermore, autoantibodies against ENO1 may serve as a promising biomarker for non-small-cell lung carcinoma [27]. ENO1 is also over-expressed in head and neck cancer [28]. It has been reported that ENO1 is involved in hypoxic. Hypoxia is a central player in cancer progression, affecting not only tumour cell autonomous functions, such as cell division and invasion, the resistance to therapy, and genetic instability, but also non-autonomous processes, such as angiogenesis and inflammation, all of which contribute to metastasis [29]. In the present study, ENO1 was found to be citrullinated in many of these cell lines. These results suggest that the high expression and citrullination of ENO1 may be involved in hypoxic and oxidative stress of tumours, thereby contributing to cancer progression.

HSP60 is a mitochondrial chaperonin that is typically responsible for the transportation and refolding of proteins from the cytoplasm into the mitochondrial matrix. In addition to its role as a heat shock protein, HSP60 functions as a chaperonin, assisting in the folding of linear amino acid chains into their respective three-dimensional structures. HSP60 has been shown to influence apoptosis in tumour cells, and this influence appears to be associated with a change in its expression level [30]. HSP60 inhibitors may function as potential anticancer agents by differentially inducing apoptosis in tumour cells [31]. The HSP60 protein is over-expressed in poorly differentiated prostate cancers [32], and HSP60 mRNA levels were significantly higher in primary breast cancer tissues compared to healthy breast tissues. Moreover, HSP60 over-expression during the first steps of breast carcinogenesis may be functionally correlated to tumour growth and/or progression [33]. In addition, cytosolic HSP60 is likely to be a regulatory component of the IkappaB kinase complex, thus implicating the first mitochondrial factor to regulate cell survival via the NF-kappaB pathway [34]. In the present study, we detected HSP60 expression in the oesophageal carcinoma cell line, cervical cancer cell line, and ovarian cancer cell line. Immunoprecipitation also indicated citrullination of the protein in various tumour cell lines. These results are in accordance with previous reports and suggest the involvement of citrullinated HSP60 in the tumorigenic process.

Unregulated migratory and invasive characteristics are common features of all cancers [35]. Microtubules are intracellular structures formed by the protein tubulin that have a number of essential cellular functions, including chromosome segregation, the maintenance of cell shape, transport, and motility, and organelle distribution. A previous genetic analysis indicated a strong association of the TUBB gene with non-small-cell lung cancer [36]. In cases of ovarian carcinoma, high expression levels of class III beta-tubulin appeared to be associated with earlier recurrence [37]. Drugs that affect the tubulin-microtubule equilibrium (Taxol and Vinca alkaloids) are effective anticancer drugs [38]. Cytokeratins are intermediate filament keratins found in the intracytoplasmic cytoskeleton of epithelial tissue. KRT8 is one of the major intermediate filament proteins expressed in single-layered epithelia of the gastrointestinal tract, and KRT8/18 expression differentiates distinct subtypes of grade 3 invasive ductal carcinoma of the breast [39]. Keratin 8/18 is broken down and reorganised during apoptosis [40]. The present study detected the expression and citrullination of TUBB in ECA, PANC-1, SKOV3, HeLa, SGC, and HEPG2 cells, as well as the expression and citrullination of KRT8 in ECA, PANC-1, SKOV3, HeLa, SGC, and HEPG2 cells. The above results in conjunction with previous studies suggest that the citrullination of TUBB and KRT8 may contribute to tumour growth and invasion and a poor prognosis.

## Conclusions

The current study identified novel citrullinated proteins, including ENO1, HSP60, KRT8, and TUBB, in tumour cells. Citrullination is a type of post-translation modification that leads to the alternation of enzyme activity and cell behaviour. Thus, the identification of novel citrullinated proteins in tumour cell lines is helpful to further the understanding of the tumorigenic process.

## Abbreviations

2-DE: Two-dimensional electrophoresis; 2-D WB: 2-D western blotting; ENO1: α-enolase; HSP60: Heat shock protein; IEF: Isoelectric focusing; KRT8: Cytokeratin 8; PAD: Peptidylarginine deiminases; PDI: Protein disulphide isomerise; PVDF: Polyvinylidene fluoride; TCR β: T cell receptor beta chain; TUBB: Tubulin β; VIME: Vimentin; WB: Western blotting.

## Competing interests

The authors declare that they have no competing interests.

## Authors’ contributions

XC drafted and finalised writing of the manuscript. ZJ, YC, and LW performed the 2-D WB and analysed the data. YZ and SY performed the immunoprecipitation. All authors read and approved the final manuscript.
